# Complexity of Infectious Diseases Compared With Other Medical Subspecialties

**DOI:** 10.1093/ofid/ofad463

**Published:** 2023-09-08

**Authors:** Brian Grundy, Eric Houpt

**Affiliations:** Division of Infectious Diseases, University of Colorado, Aurora, Colorado, USA; Division of Infectious Diseases and International Health, University of Virginia, Charlottesville, Virginia, USA

**Keywords:** Compensation, Complexity, Education, Guidelines, UpToDate

## Abstract

We aimed to highlight the complexity of the field of clinical infectious diseases compared with other medical specialties. Using available metrics, the body of knowledge within clinical infectious diseases is comparatively large and complex. This increasing complexity is underappreciated by current physician compensation schemes, needs to be carefully managed to educate future physicians, and may serve as a barrier to recruitment into the field.

## Teaser

The practice of clinical infectious diseases is based on an increasingly complex knowledge base, which carries implications for compensation, recruitment, and education.

Substantial attention has been given to the low compensation and declining fellowship applications in the field of infectious diseases (ID) [1–4]. We believe that the increasing complexity of our field is another important issue that deserves attention.

The breadth and complexity of a field of medicine is admittedly difficult to quantify and will be subject to debate. We sought to compare the field of ID with endocrinology, nephrology, and rheumatology, using available metrics. We chose to compare these other specialties because they are also primarily cognitive specialties without procedures and similar in size to ID.

**Figure 1.**
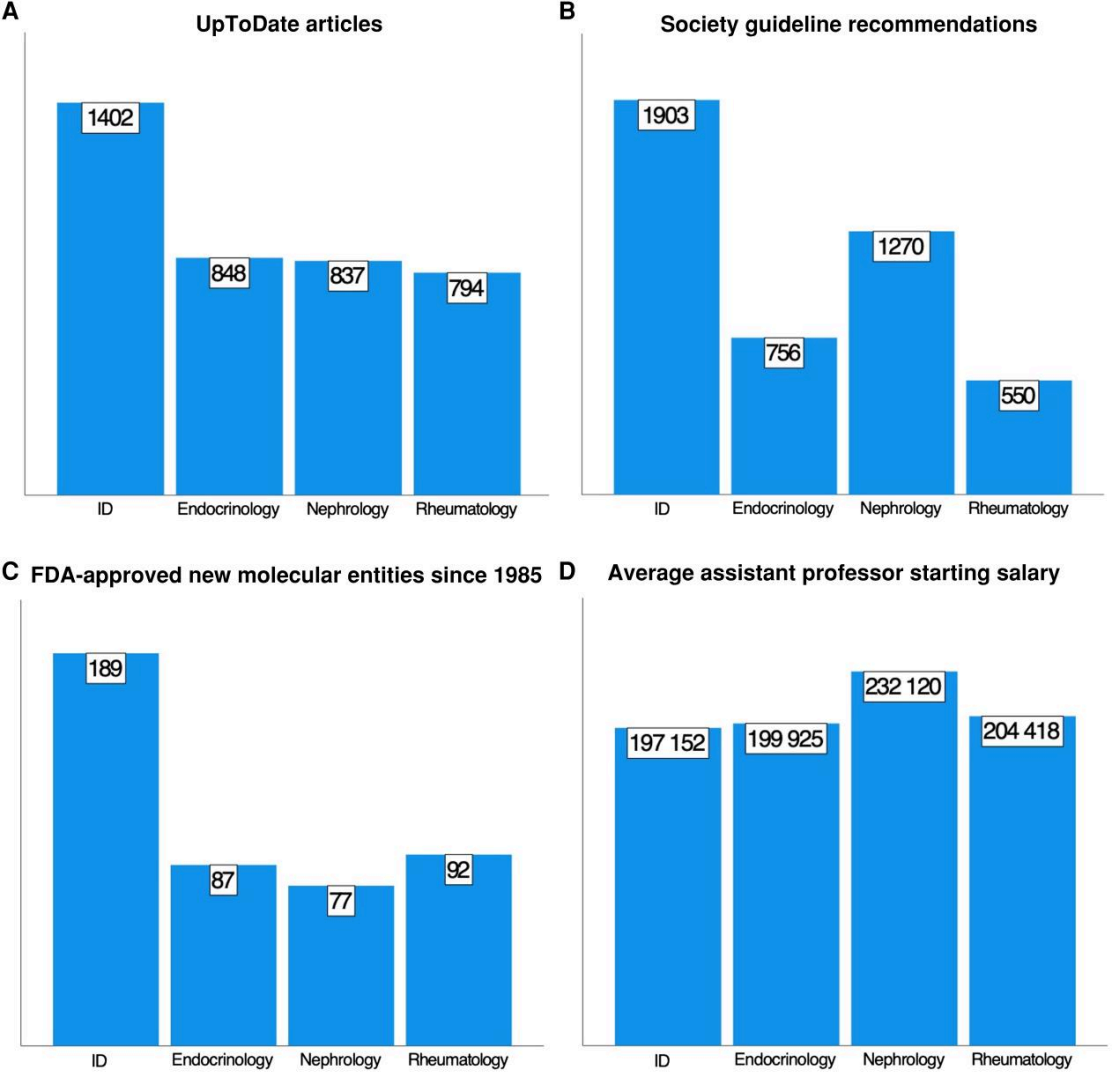
Comparison of internal medicine specialties by specialty-specific articles, society guidelines, new Food and Drug Administration (FDA)–approved medications, and starting faculty salaries. *A*, Numbers of UpToDate articles in each specialty section, as of 2 May 2023. *B*, Numbers of current evidence-based recommendations in specialty society guidelines (infectious diseases [ID], Infectious Diseases Society of America; endocrinology, Endocrine Society; nephrology, Kidney Disease: Improving Global Outcomes; and rheumatology, American College of Rheumatology). *C*, Numbers of FDA-approved new molecular entities from 1985 to 2022, for which the primary indication can be assigned to a specific specialty. *D*, Median assistant professor total compensation by specialty in 2022 (American Academy of Medical Colleges Faculty Salary Report [11]).

Since UpToDate is the most widely used clinical resource for patient care [5] we enumerated its ID content. UpToDate is organized into specialties, sections, subsections, and articles. We enumerated the number of articles across the 4 specialties. This revealed that ID contained between 65% and 77% more articles than those of the other 3 specialties (n = 1402 vs 794–848 articles; see Figure 1 and Supplementary Data for exact numbers).

Likewise, professional societies produce practice guidelines that are widely used clinical resources. Each guideline is distilled into evidence based and graded recommendations. We enumerated the number of recommendations from active guidelines (ie, excluding archived guidelines). This revealed that the Infectious Diseases Society of America (IDSA) guidelines (n = 1903 recommendations) contained between 50% and 346% more recommendations than those of the Endocrine Society, Kidney Disease: Improving Global Outcomes, and the American College of Rheumatology. We also noted that the complexity of many guidelines has increased with time. For example, the aspergillosis guidelines increased from 14 pages in 2000 to 34 pages in 2008 and to 60 pages in 2016 [6–8].

Finally, we reviewed the new molecular entities or drugs approved by the Food and Drug Administration and assigned them to specialties based on their initial approved indication. Of 1199 new entities approved by the Food and Drug Administration from 1985 through 2021 [9], 189 (16%) have an ID indication, substantially more than the number ascribable to endocrinology, nephrology, or rheumatology.

Our message is not that the fields of endocrinology, nephrology, and rheumatology are simple. Their knowledge bases are also large, and we all take care of patients with extremely complicated disease processes and comorbid conditions. Indeed, one large study found that patient complexity was highest among those seen by nephrologists and ID physicians [10]. Rather, our argument is that the knowledge base required of the ID physician is increasingly complex. For example, in just 3 years UpToDate has produced 29 articles related to coronavirus disease 2019 (COVID-19), and the IDSA has 70 pages of guidelines on COVID-19. Given this ever-evolving complexity, we see a number of important implications for our field.

First, the complexity of ID clinical decision making is not adequately appreciated or compensated by payers. The physician work relative value unit is supposed to reflect the time and intensity associated with furnishing a service; however, there is no additional intensity that can be assigned because of the complexity of our field or decision making. Notably, according to the American Academy of Medical Colleges [11], the median total compensation for an assistant professor in ID is less than for the other specialties (Figure 1). The IDSA is well aware of this and has authored letters to Congress stating that ID reimbursement does not adequately reflect the value and complexity of care provided by ID clinicians [12]. In other fields such as business, law, and engineering, experts that command broad and deep expertise will generally receive greater compensation. We hope data such as these can be used to advocate for the complexity of our decision making and enhanced compensation.

Second, while the complexity of the field of ID may be appealing for some of us that entered the specialty, it may present an unattractive lifestyle choice for others, particularly when coupled with the low compensation of the field. Difficulty in mastering a field has been noted as a reason for declining interest in ID [13]. While the high complexity of our field cannot be reduced, we need to acknowledge it and identify ways to recruit in the face of it [14].

Third, how to educate future and current physicians on the large and increasing content of ID will become increasingly difficult. This affects not only our ID fellows but also medical residents and students who do not specialize in ID. The primary pedagogical method for ID material has been didactics and memorization [1]. This will become more difficult. For the 80% of US counties that do not have an ID physician already [15] general practitioners will need to manage infections amidst this increasing content. How to distill knowledge and guidelines to generalists will become increasingly challenging and important.

Fourth, how we practice as ID divisions and subspecialty groups may need to evolve. It will become harder for the general ID specialist to stay abreast of the latest information and evidence in all areas. Large ID groups and divisions may need to further develop sub-subspecialists within ID for optimal clinical care, much as oncology does.

In summary, the high complexity of the ID field is a reality and will likely increase. We believe that this complexity is inadequately captured by current compensation schemes and is likely a contributor to the declining applicant pool. The increasing body of ID knowledge content coupled with a flat or declining number of specialists is an ominous formula. These systemic trends will likely continue, and clinical care stands to worsen without significant structural reform.

## Supplementary Material

ofad463_Supplementary_Data

## Supplementary Data

Supplementary materials are available at *Open Forum Infectious Diseases* online. Consisting of data provided by the authors to benefit the reader, the posted materials are not copyedited and are the sole responsibility of the authors, so questions or comments should be addressed to the corresponding author.

## References

[1] Bonura EM , Lee ES, Ramsey K, Armstrong WS. Factors influencing internal medicine resident choice of infectious diseases or other specialties: a national cross-sectional study. Clin Infect Dis 2016; 63:155–63. 27126345 10.1093/cid/ciw263 PMC4928385

[2] Sullivan T . Declining interest in infectious diseases: an economic problem that requires economic solutions. Clin Infect Dis 2016; 63:1677. 10.1093/cid/ciw671 27682071

[3] Barsoumian AE , Hartzell JD, Bonura EM, Ressner RA, Whitman TJ, Yun HC. Factors influencing selection of infectious diseases training for military internal medicine residents. Clin Infect Dis 2018; 67:1582–7. 29912315 10.1093/cid/ciy322

[4] Mohareb AM , Brown TS. Medical student debt and the US infectious diseases workforce. Clin Infect Dis 2022; 76:1322–7. 10.1093/cid/ciac862 PMC10396319 36318609

[5] De Leo G , LeRouge C, Ceriani C, Niederman F. Websites most frequently used by physician for gathering medical information. AMIA Annu Symp Proc 2006; 2006:902. 17238521 PMC1839616

[6] Stevens DA , Kan VL, Judson MA, et al Practice guidelines for diseases caused by *Aspergillus*. Clin Infect Dis 2000; 30:696–709. 10770732 10.1086/313756

[7] Walsh TJ , Anaissie EJ, Denning DW, et al Treatment of aspergillosis: clinical practice guidelines of the Infectious Diseases Society of America. Clin Infect Dis 2008; 46:327–60. 18177225 10.1086/525258

[8] Patterson TF , Thompson GR III, Denning DW, et al Practice guidelines for the diagnosis and management of aspergillosis: 2016 update by the Infectious Diseases Society of America. Clin Infect Dis 2016; 63:e1–e60. 27365388 10.1093/cid/ciw326 PMC4967602

[9] US Food and Drug Administration . Compilation of CDER NME and new biologic approvals 1985–2022. 2023. Available at: https://www.fda.gov/drugs/drug-approvals-and-databases/compilation-cder-new-molecular-entity-nme-drug-and-new-biologic-approvals. Accessed 2 May 2023.

[10] Tonelli M , Wiebe N, Manns BJ, et al Comparison of the complexity of patients seen by different medical subspecialists in a universal health care system. JAMA Netw Open 2018; 1:e184852. 30646392 10.1001/jamanetworkopen.2018.4852 PMC6324421

[11] American Academy of Medical Colleges . AAMC Faculty Salary Report FY 2022. 2023. Available at: https://store.aamc.org/aamc-faculty-salary-report-fy-2022-online.html. Accessed 2 May 2023.

[12] Walensky RP , Del Rio C, Armstrong WS. Charting the future of infectious disease: anticipating and addressing the supply and demand mismatch. Clin Infect Dis 2017; 64:1299–301. 28387806 10.1093/cid/cix173

[13] Chandrasekar P , Havlichek D, Johnson LB. Infectious diseases subspecialty: declining demand challenges and opportunities. Clin Infect Dis 2014; 59:1593–8. 25148890 10.1093/cid/ciu656 PMC7108050

[14] Cutrell JB . #WhyID: crowdsourcing the top reasons to choose infectious diseases in the age of twitter. Open Forum Infect Dis 2019; 6:ofz403. 31660365 10.1093/ofid/ofz403 PMC6785704

[15] Bono RS , Dahman B, Sabik LM, et al Human immunodeficiency virus–experienced clinician workforce capacity: urban-rural disparities in the Southern United States. Clin Infect Dis 2021; 72:1615–22. 32211757 10.1093/cid/ciaa300 PMC8096280

